# Enhancing meningitis diagnosis: a diagnostic stewardship workflow in a resource-limited setting

**DOI:** 10.1128/spectrum.04173-25

**Published:** 2026-08-04

**Authors:** Alfredo Chiappe Gonzalez, Juan Carlos Gómez de la Torre, Juan José Montenegro-Idrogo, Nicanor Mori, Evelyn Villacorta, Lizzet Martínez-Dionisio, Carolina Cucho, Luis Alvarado, Renee Newby, Joseph Zunt, Jaime Soria

**Affiliations:** 1 Instituto de Investigaciones en Ciencias Biomédicas, Universidad Ricardo Palma, Lima, Peru; 2 Hospital Nacional Dos de Mayo 504674, Lima, Peru; 3 Northern Pacific Global Health Research Fellows Training Consortium, University of Washington 7284, Seattle, Washington, USA; 4 Facultad de Medicina, Universidad de Piura, Lima, Peru; 5 ROE Laboratorio Clínico, Lima, Peru; 6 Facultad de Ciencias de la Salud, Universidad Científica del Sur, Lima, Peru; 7 Hospital Nacional Daniel A. Carrion 269034, Callao, Peru; 8 Division of Infectious Diseases, University of Kentucky 4530, Lexington, Kentucky, USA; 9 Hospital Maria Auxiliadora, Lima, Peru; 10 Department of Neurology, University of Washington 7284, Seattle, Washington, USA; 11 Department of Global Health, University of Washington, Seattle, Washington, USA; 12 Department of Medicine (Infectious Diseases), University of Washington, Seattle, Washington, USA; 13 Department of Epidemiology, University of Washington, Seattle, Washington, USA; Laboratory Corporation of America Holdings, Burlington, North Carolina, USA

**Keywords:** meningitis, diagnosis, stewardship, molecular test

## Abstract

Central nervous system (CNS) infections cause high morbidity and mortality, especially in low- and middle-income countries, where the burden is unevenly distributed. This study aims to develop a diagnostic stewardship workflow (DSW) to improve diagnostic efficiency and increase pathogen detection in resource-limited settings. We conducted a prospective cross-sectional study in adults with suspected meningitis in Lima, Peru. Cerebrospinal fluid specimens were processed in parallel by the hospital laboratory (standard of care [SoC]) and an independent reference laboratory executing the DSW, enabling within-patient head-to-head comparison. The DSW was compared with the SoC using McNemar’s and Cochran’s Q tests with Holm–Bonferroni correction. Among 103 adults (median age: 39 years, 36.9% HIV-positive), CNS infection was confirmed in 44 (42.7%), and a non-infectious etiology was diagnosed in 20 (19.4%). The DSW achieved significantly higher pathogen detection rates (97.7%; 43/44 confirmed cases). Incremental yield over SoC was etiology-specific: 0 pp for cryptococcosis, +36 pp for tuberculous meningitis, +67 pp for bacterial meningitis, and +100 pp for viral encephalitis. A deterministic cost simulation showed that applying the DSW with sequential stopping rules would reduce per-cohort diagnostic expenditure by approximately 61% compared with the all-tests-to-all strategy applied in this study. A DSW substantially increases pathogen detection compared to standard diagnostic procedures, with the largest incremental gains for viral and difficult-to-culture bacterial pathogens, while reducing unnecessary testing and diagnostic costs and providing practical guidance to clinicians to streamline the diagnostic process.

**IMPORTANCE:**

We developed a meningitis diagnostic stewardship workflow using clinical information and initial test results to guide decision-making and streamline pathogen detection. This approach increased diagnostic yield, has the potential to reduce unnecessary testing, and may support more efficient diagnostic care in resource-limited settings.

## INTRODUCTION

Central nervous system (CNS) infections are associated with high morbidity and mortality. Meningitis ranks among the top four neurological disorders related to the highest disability-adjusted life years (1). The global estimate of the burden of CNS infections is difficult to calculate due to the scarcity of population-based data, the heterogeneity of pathogens causing CNS infections, and underreporting. In addition, the burden of CNS infections is unevenly distributed, especially in low- and middle-income countries (LMICs). Sub-Saharan Africa has the highest prevalence and mortality rates associated with CNS infections. In people with HIV infection, CNS infections represent 15%–25% of all causes of mortality (2).

The most common etiologies of CNS infection are viral, bacterial, cryptococcal, and tuberculosis (TB). Previous studies on acute meningitis in South America have predominantly reported herpesvirus and bacterial infections, whereas chronic meningitis was most often due to *Mycobacterium tuberculosis* and *Cryptococcus* spp. (3, 4). Particularly relevant to LMICs, TB remains a common and persistent public health problem. The World Health Organization (WHO) estimated that more than 10 million people develop TB every year, which is the second most common cause of death due to a single infectious agent after SARS-CoV-2 infection in 2022. Globally, 7.5 million people were newly diagnosed with TB during the year 2022; approximately 2% of these infections involved the CNS, most often manifesting as meningitis or tuberculous granuloma (5). Mortality due to TB meningitis (TBM) is around 50% in low-income settings (6). Cryptococcal meningitis (CM) is a frequent etiology of CNS infection in people with HIV infection and a common cause of death in many resource-limited countries, with mortality rates as high as 100% (7). Similarly, bacterial meningitis (BM), which causes high mortality, is also frequent, especially in resource-limited settings (8).

The most significant limitation to the timely administration of appropriate antimicrobial treatment for CNS infections remains the determination of the etiology. While newer diagnostic methods promise to increase the speed and accuracy of identifying pathogens in cerebrospinal fluid (CSF), an etiologic pathogen is detected in less than 50% of encephalitis cases (9, 10). Given the diagnostic limitations, a different strategic approach to diagnosing meningitis is needed to accompany the rapid development of new diagnostic technologies, especially in developing countries, where cost-effective molecular tools are required for more efficient healthcare (11, 12). In Peru, routine diagnostics rely heavily on slow mycobacterial culture, inconsistent availability of cryptococcal antigen tests, and limited access to molecular assays. This combination leads to delayed pathogen identification in many public hospitals, including ours.

This study aims to develop a diagnostic stewardship workflow (DSW) that improves diagnostic efficiency, reduces unnecessary testing, and increases pathogen detection in a resource-limited setting and compare its diagnostic yield with the institutional standard of care (SoC) in a head-to-head, parallel-laboratory design, including a deterministic cost simulation in local currency.

## MATERIALS AND METHODS

### Study design and data collection

We conducted a prospective cross-sectional study of adults with suspected meningitis admitted to the Hospital Nacional Dos de Mayo in Lima, Peru, between 2018 and 2019. Patients identified in the emergency department, internal medicine, neurology, and infectious disease wards who had neurological symptoms consistent with meningitis (nuchal rigidity, Brudzinski’s sign, Kernig’s sign, headache, nausea, or altered mental status) and who required a diagnostic lumbar puncture were enrolled. Eligible patients were 18 years of age or older, not pregnant, not currently taking antibiotics, and able to speak Spanish or English. The patient or their legal representative consented to participate in the study, and a lumbar puncture was carried out by the treating physician, an infectious diseases consultant, or a neurologist as part of the usual workup.

We collected clinical data and the results of standard microbiologic studies performed at the hospital. All data collected were managed using REDCap electronic data capture tools hosted at the University of Washington. Each enrolled patient contributed cerebrospinal fluid that was simultaneously processed by two laboratories under a parallel-testing design: the hospital laboratory of Hospital Nacional Dos de Mayo (HNDM/MINSA), which performed the institutional standard-of-care menu, and the reference laboratory (a private reference laboratory), which executed the diagnostic stewardship workflow. The SoC menu comprised CSF cytochemistry, Gram stain, India ink, Ziehl-Neelsen smear, bacterial and fungal cultures, mycobacterial culture in Ogawa medium, GeneXpert MTB/RIF when clinically requested, and VDRL on request. The DSW menu comprised the same CSF cytochemistry and direct stains, latex agglutination for cryptococcal and bacterial antigens, lateral flow CrAg, universal VDRL, mycobacterial culture in Löwenstein–Jensen medium, Xpert MTB/RIF Ultra performed systematically on every sample, and the BioFire FilmArray Meningitis/Encephalitis panel. The same patient-level CSF specimens were evaluated under both diagnostic menus, allowing within-patient pairwise comparisons of detection. Because some SoC assays were performed only when clinically requested, sample sizes differ across tests; DSW assays were performed systematically on available CSF samples.

### Laboratory workflow and procedures

A total of 10–20 mL of CSF was collected through lumbar puncture; lateral flow assay (LFA) was performed by adding 40 µL of CSF to one drop of CSF diluent, which was incubated for 10 min with the CrAg LFA test strip, according to CrAg LFA IMMY manufacturer instructions. A CSF transport container with 10 mL was sent to a specialized laboratory for research purposes, and four cryovials of 2–3 mL each were sent to the hospital laboratory for standard-of-care management. Standard meningitis tests included Gram and India ink stains, Ziehl-Neelsen smear, mycobacterial (Ogawa), bacterial, and fungal cultures.

**Fig 1:**
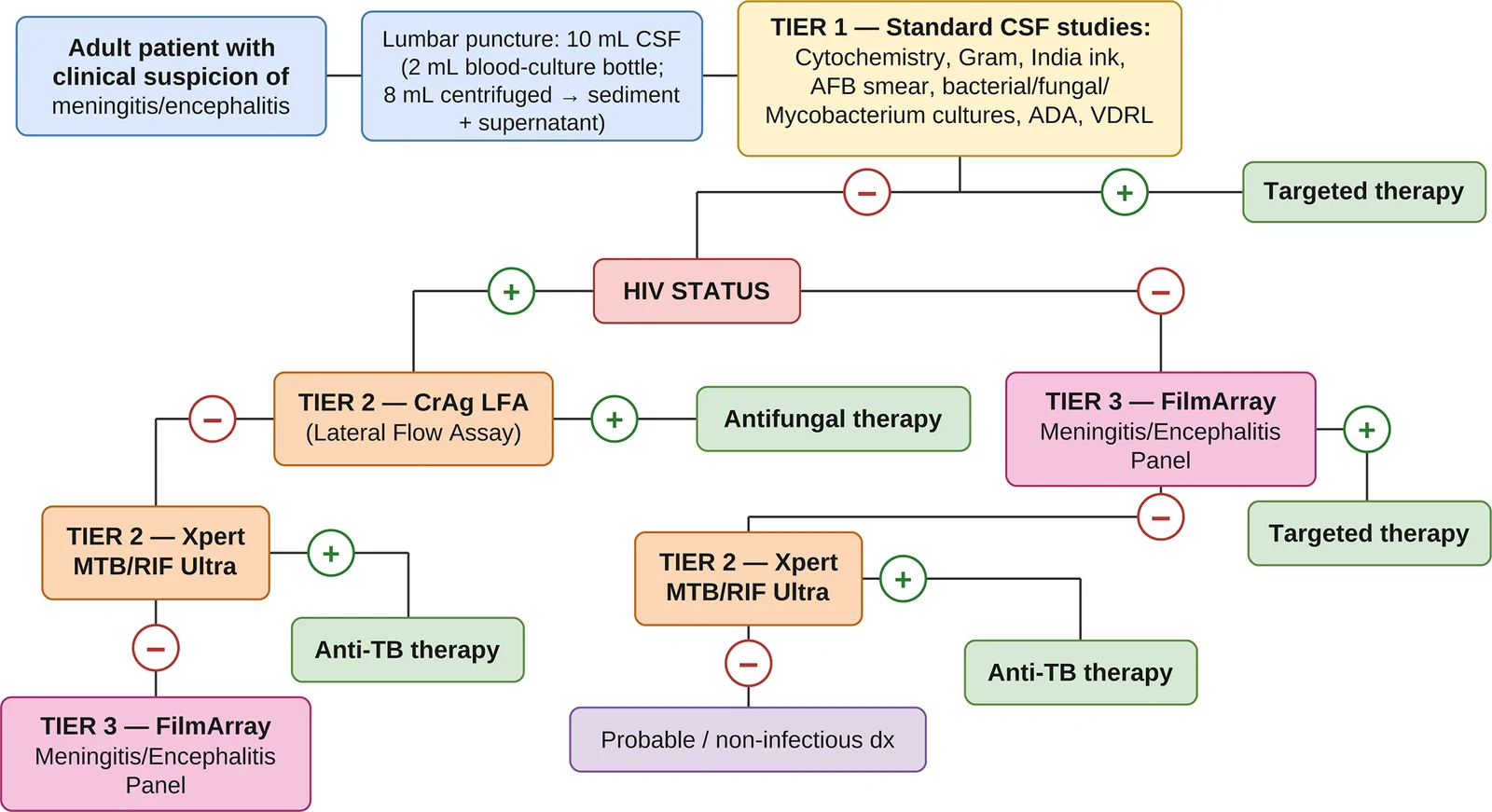
Diagnostic stewardship workflow for suspected meningitis (adults). The algorithm proceeds in three sequential tiers with explicit stopping rules: Tier 1—basic SoC tests (cytochemistry, Gram, India ink, AFB smear, basic cultures, and adenosine deaminase [ADA]); Tier 2—applied if prior tiers negative (Xpert MTB/RIF Ultra and CrAg LFA); Tier 3—applied if prior tiers negative (BioFire FilmArray ME Panel). At each node, a positive result halts further testing and triggers directed therapy; a negative result advances the patient to the next tier. HIV status branches the workflow toward cryptococcal-priority (HIV+) versus bacterial-priority (HIV−) testing, so that in HIV-negative patients the Tier 3 ME Panel precedes Tier 2 Xpert MTB/RIF Ultra.

To evaluate the incremental diagnostic yield of a structured algorithm, each CSF specimen obtained from a single lumbar puncture was split and processed in parallel by two independent laboratories: the Hospital Nacional Dos de Mayo laboratory (SoC) and ROE, a reference private laboratory (DSW). This was a within-patient comparison of two diagnostic menus, not a like-for-like comparison of identical assays. The complete diagnostic algorithm, including sequential decision nodes, stopping rules, and the laboratory of origin for each test, is presented in Fig. 1.

Of the 10 mL received at the reference laboratory, 200 μL was removed for molecular detection of pathogens using the FilmArray technique with the BioFire FilmArray Meningitis/Encephalitis (ME) Panel, according to the manufacturer’s instructions. This method automates the extraction of genetic material from a cartridge with multiple compartments. The extracted genetic material is reverse-transcribed to cDNA and amplified by PCR using specific primers targeting molecular targets (*Escherichia coli*, *Haemophilus influenzae*, *Listeria monocytogenes*, *Neisseria meningitidis*, *Streptococcus agalactiae*, *Streptococcus pneumoniae*, Cytomegalovirus, *Cryptococcus neoformans*, enterovirus, herpes simplex virus 1 and 2, human herpesvirus 6, human parechovirus, and varicella-zoster virus). Amplicons were amplified in a second stage in an “array” with the second pair of primers specific to each pathogen. Fluorescence emitted by SYBR green probes recognizes an amplified molecular target. Next, 2 mL of CSF was placed in one BACTEC Plus Aerobic/F medium blood culture bottle. Similarly, 0.5 mL was run through an automated hematology counter to determine leukocyte count and cell type differentiation. The remaining sample was centrifuged at 1,000*g* for 10 min in an Eppendorf centrifuge 5702, after which the supernatant was pipetted into four 5 mL sterile tubes using a sterile Pasteur pipette. Using the supernatant, we performed agglutination tests for *Cryptococcus* spp. with the IMMY Latex-Cryptococcus Antigen Detection System according to the manufacturer’s instructions. The VDRL method was performed to determine the presence of syphilis reagins.

Aliquots from the CSF sediment were used for Gram and India ink stains and Ziehl-Neelsen smear. Mycobacterial, bacterial, and fungal cultures were performed in solid culture media: MacConkey agar, sheep blood agar prepared with tryptic soy agar, and Sabouraud dextrose agar. For *Mycobacterium*, Löwenstein–Jensen medium was used. From the remaining sediment, molecular testing using the GeneXpert Xpert MTB/RIF Ultra cartridge was performed to detect the genetic material of *Mycobacterium tuberculosis*. A volume of 0.5 mL of sediment was added to 1.5 mL of diluent, followed by automated extraction of genetic material and amplification of three molecular targets (rpoB, IS6110, and IS1081) of the genome of the *Mycobacterium tuberculosis* complex. Real-time polymerase chain reaction used seven specific fluorescence probes targeting rpoB (5), IS6110 (1), and IS1081 (1) for amplicon recognition via melting temperature differences. Glucose, protein, and lactate dehydrogenase (LDH) levels were determined using automated biochemistry equipment (Roche Cobas 6002) with a glucose reagent based on the hexokinase method. LDH was evaluated using L-lactate conversion to pyruvate, and total protein was determined using turbidimetry with EDTA and benzethonium chloride. Adenosine deaminase (ADA) activity was measured in the supernatant using the Giusti colorimetric method.

### Case adjudication and etiologic definitions

Final etiologic categorization was performed by a clinical adjudication committee (ACG, JJM, JS, and NM) at hospital discharge, using composite criteria applied to the entire course of the admission. Confirmed infection was defined as direct microbiological detection of an etiologic agent by Xpert MTB/RIF or Xpert MTB/RIF Ultra, mycobacterial culture, fungal or bacterial culture, lateral flow CrAg, latex agglutination, or BioFire FilmArray ME, which was both consistent with the clinical syndrome and supported by therapeutic response during admission for bacterial and viral cases. For bacterial and viral diagnoses, molecular positivity alone was insufficient: clinical–CSF concordance and clinical improvement with targeted therapy were required to mitigate the well-recognized risk of FilmArray false positives due to low pathogen burden or contamination. Probable infection was defined by a clinical syndrome consistent with meningitis, compatible CSF cytochemistry (pleocytosis, low glucose, and elevated protein) and/or elevated ADA, decision by the treating team to initiate targeted antimicrobial therapy, and clinical improvement on therapy through discharge in the absence of confirmatory microbiology. Non-infectious diagnosis required identification of an alternative cause (e.g., autoimmune CNS disease, status epilepticus, and structural cerebrovascular event) with documented response to non-antimicrobial therapy. The committee was not blinded to laboratory results because longitudinal clinical adjudication necessarily integrates microbiology with the clinical course; however, all category assignments were finalized only at discharge, after observation of treatment response, to minimize the risk of premature reliance on molecular signals.

### Statistical analysis

Descriptive statistics were conducted for clinical and epidemiological characteristics. Kruskal–Wallis and Fisher’s exact tests were used to detect overall differences among the diagnostic groups. Multiple pairwise comparisons among the final diagnosis categories were performed; the Bonferroni adjustment was used for categorical variables, while Dunn’s test was performed for continuous variables. Additionally, we used a multinomial logistic regression model to examine the relationship between potential predictor variables and the diagnostic categories, using one category (“non-infectious etiology”) as the reference; the model estimates the relative risk ratio (RRR). The delta method estimates differences in predicted probabilities between each pair of diagnostic categories.

A classification tree model was developed using recursive partitioning. The Gini impurity index was used as the splitting criterion to determine the most informative variables for stratifying patients into diagnostic categories. HIV status was selected as the first-tier classifier due to its significant influence on meningitis etiology and infection risk. The anticipated outcome was a data-driven approach to develop a DSW algorithm for patients with meningitis and compare its diagnostic performance with the local standard of care. McNemar’s and Cochran’s Q tests were used to compare diagnostic tests pairwise; for all pairwise McNemar comparisons of test pairs reported in Tables 1 and 2, *P* values were adjusted using the Holm–Bonferroni method applied separately within two families to control family-wise error at *α* = 0.05: the seven paired test-level comparisons in Table 1, and the five patient-level pathogen-detection comparisons in Table 2. The decision logic of the DSW, including the role of HIV testing as the first stratifier, is summarized in Fig. 1. STATA (StataCorp, version 17.0, College Station, TX, USA) was used for all statistical analyses.

**TABLE 1:** Positive tests by laboratory location, with paired comparison

Test	Standard (samples)^*a*^	Standard (positives)	Study (samples)	Study (positives)	Paired *n*	Raw *P^b^*	Holm-adj *P^b^*
Gram stain	99	4	99	13	95	0.021	0.150
Bacterial culture	93	4	99	6	90	0.625	1.000
India ink	103	12	100	13	100	1.000	1.000
Fungal culture	75	8	100	14	75	0.500	1.000
Latex CrAg (study only)	–^*c*^	–	103	14	–	NA^*d*^	NA
Lateral flow CrAg (study only)	–	–	103	13	–	NA	NA
Ziehl–Neelsen stain	103	1	99	1	99	1.000	1.000
Mycobacterial culture (Ogawa)	47	5	99	10	47	0.500	1.000
Xpert MTB/RIF (standard) vs Xpert Ultra (study)	27	4	99	13	25	1.000	1.000
ME panel—bacterial targets (study only)	–	–	103	13	–	NA	NA
ME panel—viral targets (study only)	–	–	103	4	–	NA	NA
Pathogens identified^*e*^	103	26	103	43		–	–

^*a*^ Sample sizes (Samples columns) differ across tests because some assays were performed only on clinical request in the standard-of-care arm (e.g., GeneXpert MTB/RIF, Ogawa culture), while in the DSW arm, the same assays were performed systematically on every available CSF sample. The columns “Samples” include all samples with a recorded result for that assay, including negative and positive results. The column Positives reports the number of positive results among tested samples.

^*b*^ The raw *P* values were calculated using the exact McNemar test based on patient-level paired data; only patients who underwent testing in both laboratories were included in each comparison. Holm–Bonferroni adjustment was applied to the family of paired test-level comparisons (*n* = 7).

^*c*^ –, not applicable.

^*d*^ No paired comparison possible because the test was performed only at one laboratory. ME, meningitis/encephalitis panel.

^*e*^ Overall diagnostic yield (“Pathogens identified”) is reported descriptively in this table; the formal paired comparison appears in Table 2.

**TABLE 2:** Diagnostic yield and per-pathogen concordance between standard of care (HNDM/MINSA) and diagnostic stewardship workflow (ROE) among confirmed cases (*n* = 44)^*a*^

Etiology	Confirmed (*n*)	Both labs positive	Only SoC positive	Only DSW positive	DSW vs SoC absolute Δ	Raw *P* (exact McNemar)	Holm-adj *P*
Cryptococcus	14	14	0	0	0 pp	1.000	1.000
Tuberculosis	14	7	1	6	+36 pp	0.125	0.375
Bacterial	12	4	0	8	+67 pp	0.008	0.031
Viral	4	0	0	4	+100 pp	0.125	0.375
Overall	44	25	1	18	+39 pp	<0.001	<0.001

^*a*^ Paired exact McNemar tests were applied within each etiology stratum. Holm–Bonferroni adjustment within the family of five comparisons (four etiology subgroups + overall). Absolute differences are reported in percentage points. DSW, diagnostic stewardship workflow; SoC, standard of care.

### Cost analysis

Cost simulation was based on the institutional tariffs of the Peruvian public system reference laboratory for 2018–2019 (Table S1). Because every patient received every assay in both menus during the study (parallel testing without real-time stopping rules), the cost simulation was a counterfactual exercise that asked how much expenditure could have been avoided had the DSW been applied with sequential stopping rules. The workflow was modeled as a sequential algorithm in which all patients receive Tier-1 testing (unit cost S/. 599.95); only Tier-1-negative patients proceed to Tier-2 (incremental cost S/. 507.00 for Xpert MTB/RIF Ultra); and only Tier-2-negative patients proceed to Tier-3 (incremental cost S/. 1,625.00 for FilmArray ME). Two scenarios were compared: (i) the stopping-rule DSW, in which testing halts at the first positive tier; and (ii) an all-tests-to-all comparator in which every patient receives the complete battery, reflecting universal application of the expanded DSW menu as conducted in this study. We computed costs for both the 44 confirmed cases and the full 103-patient analytical cohort to bound the upper and lower estimates of savings. Per-test tariffs and the full cost matrix are provided in Table S1.

To assess the robustness of the savings estimate to uncertainty in the workflow-level distribution, we recomputed the total cost under ±20% variation in the proportion of patients resolved at Tier 1. Cost savings remained within 54.7%–69.3% among confirmed cases and 23.4%–29.6% across the full 103-patient cohort, indicating that the dominant economic driver (avoiding FilmArray in patients resolved at Tier 1 or 2) is insensitive to ±20% variation in stratification.

## RESULTS

The study enrolled 106 adult patients with clinical features of meningitis. However, three patients were excluded due to insufficient data. We confirmed CNS infection in 44 (42.7%) patients. Case definitions for the confirmed, probable, and non-infectious categories are detailed in Case adjudication and etiologic definitions in Materials and Methods, in which *M. tuberculosis* was identified in 14, *C. neoformans* in 14, bacteria in 12, and a viral agent in 4. A non-infectious etiology was diagnosed in 20 (19.4%) patients, and 39 (37.9%) patients had a probable infectious etiology.

Most patients were male (72.8%), with a median age of 39 years (IQR: 28–58); 38 (36.9%) patients had a diagnosis of HIV, most of them with a detectable viral load (>400 copies/mL), and only 11 patients were taking antiretroviral therapy.

Among patients with cryptococcal meningitis, 11/14 (78.6%) were HIV-positive (RRR: 10.9; 95% CI: 2.16–56.09; *P* = 0.004). In tuberculous meningitis, 6/14 (42.9%) were HIV-positive, though this association was not statistically significant (RRR: 2.3; 95% CI: 0.52–9.73; *P* = 0.278) (Table 3).

**TABLE 3:** Demographic and clinical characteristics of patients with suspected meningitis by etiological diagnosis

Characteristics^*a*^	Non-infectious (*n* = 20)	Probable infection (*n* = 39)	Tuberculosis (*n* = 14)	Cryptococcal (*n* = 14)	Bacterial (*n* = 12)	Viral (*n* = 4)	*P* value
Age (years)	38 (25–68)	38 (28–55)	36 (21–51)	39 (30–50)	59 (48–64)	31 (25–43)	0.218
Male sex	14 (70.0%)	28 (71.8%)	10 (71.4%)	13 (92.9%)	6 (50.0%)	4 (100.0%)	0.198
HIV infection	5 (25.0%)	13 (33.3%)	6 (42.9%)	11 (78.6%)	0 (0.0%)	3 (75.0%)	<0.001
CD4 < 200	4/5 (80.0%)	11/13 (84.6%)	3/5 (60.0%)	8/8 (100.0%)	NA^*b*^	3/3 (100.0%)	0.343
Viral load > 400	5/5 (100%)	12/13 (92.3%)	4/5 (80.0%)	7/8 (87.5%)	NA^*b*^	3/3 (100.0%)	0.896
Antiretroviral therapy	2/5 (40.0%)	4/13 (30.8%)	3/6 (50.0%)	2/11 (18.2%)	NA^*b*^	0 (0.0%)	0.561
Prior tuberculosis	1 (5.0%)	5 (12.8%)	4 (28.6%)	2 (14.3%)	1 (8.3%)	1 (25.0%)	0.282
Diabetes mellitus	1 (5.0%)	4 (10.3%)	0 (0.0%)	1 (7.1%)	3 (25.0%)	0 (0.0%)	0.387
Symptom duration (days)	7 (4–18)	7 (3–15)	7 (7–15)	12 (7–30)	3 (2–11)	12 (4–100)	0.164
Weight loss	5 (25.0%)	15 (38.5%)	9 (64.3%)	7 (50.0%)	2 (16.7%)	4 (100.0%)	0.012
Headache	14 (70.0%)	29 (74.3%)	14 (100.0%)	14 (100.0%)	12 (100.0%)	3 (75.0%)	0.011
Fever	11 (55.0%)	30 (76.9%)	9 (64.3%)	9 (64.3%)	8 (66.7%)	3 (75.0%)	0.647
Altered level of consciousness	13 (65.0%)	27 (69.2%)	11 (78.6%)	10 (71.4%)	11 (91.7%)	3 (75.0%)	0.655
Focal neurologic deficits	6 (30.0%)	7 (18.0%)	5 (35.7%)	2 (14.3%)	2 (16.7%)	1 (25.0%)	0.635
Neck stiffness	10 (50.0%)	16 (41.0%)	11 (78.6%)	9 (64.3%)	11 (91.7%)	2 (50.0%)	0.014
Kernig’s sign	7 (35.0%)	12 (30.8%)	7 (50.0%)	8 (57.1%)	3 (25.0%)	2 (50.0%)	0.399

^*a*^ Values are Median (IQR) or *n* (%).

^*b*^ NA, not applicable.

Meningitis symptoms at presentation were present for a median of 7 days (IQR: 4–15). The most common symptoms were headache (83.5%), altered level of consciousness (72.8%), and fever (67.9%). The most common physical examination finding was neck stiffness (57.2%), and 23 (22.3%) patients had focal neurological signs. Weight loss was significantly associated with tuberculosis (RRR: 5.4; 95% CI: 1.21–23.96; *P* = 0.027), neck stiffness was significantly associated with BM (RRR: 11.0; 95% CI: 1.18–101.97; *P* = 0.035), and symptoms of intracranial hypertension were associated with cryptococcal meningitis (RRR: 10.0; 95% CI: 2.02–49.29; *P* = 0.005) (Table 3).

The CSF opening pressure was reported in 89 patients, with a median of 23 cm H_2_O (IQR: 11–30). Increased CSF opening pressure (>200 mm H_2_O) was found in 48 (53.9%) patients and was mainly associated with BM (RRR: 8.1; 95% CI: 1.23–53.20; *P* = 0.029). Pleocytosis was present in 67 (65.1%) patients, and CSF white blood cell counts were significantly higher in BM than in other causes. CSF protein, ADA, and LDH were increased in TBM and BM; glucose was decreased significantly in bacterial infections (Table 4).

**TABLE 4:** CSF characteristics of patients with suspected meningitis by etiological diagnosis

CSF characteristics	Non-infectious (*n* = 20)	Probable infection (*n* = 39)	Tuberculosis (*n* = 14)	Cryptococcal (*n* = 14)	Bacterial (*n* = 12)	Viral (*n* = 4)	*P* value
Opening CSF pressure (cm H2O)^*a*^	16 (8–30)	18 (10–28)	23 (20–28)	28 (19–43)	30 (25–35)	29 (19–33)	0.04
Intracranial hypertension	5/14 (35.7%)	13/34 (38.2%)	9/13 (69.2%)	9/13 (69.3%)	9/11 (81.8%)	3 (75.0%)	0.036
White blood cells (cells/µL)^*a*^	2 (1–7)	20 (2–75)	63 (18–163)	25 (3–43)	316 (163–572)	28 (0–133)	0.001
Protein (mg/dL)^*a*^	60 (30–92)	70 (40–162)	224 (130–310)	70 (44–207)	587 (426–802)	154 (130–220)	<0.001
Protein >45 mg/dL	13 (65.0%)	28 (71.8%)	14 (100.0%)	10 (71.4%)	12 (100.0%)	4 (100.0%)	0.023
Glucose (mg/dL)^*a*^	56 (48–71)	57 (47–79)	29 (15–37)	37 (10–44)	8 (2–50)	55 (44–65)	<0.001
Glucose <45 mg/dL	3 (15.0%)	8 (20.5%)	13 (92.9%)	11 (78.6%)	9 (75.0%)	1 (25.0%)	<0.001
LDH (U/L)^*a*^	29 (20–42)	26 (20–50)	59 (52–194)	34 (28–109)	280 (128–450)	84 (46–203)	<0.001
ADA (U/L)^*a*^	2.5 (1–3.9)	2.6 (1.3–5.3)	6.7 (3.3–8.0)	3.7 (1.6–6.0)	6.2 (4.5–14.2)	4.4 (2.6–9.3)	0.002
ADA > 9 (U/L)	0 (0.0%)	3/37 (8.1%)	2 (14.3%)	0/13 (0.0%)	4/12 (33.3%)	1 (25.0%)	0.025

^*a*^ Median (IQR); LDH, lactate dehydrogenase; ADA, adenosine deaminase.

Regarding the confirmed cases, *M. tuberculosis* was identified in 1/14 by Ziehl-Neelsen stain, in 13/13 by PCR, and in 5/8 by culture in solid media, with a median growth time of 10 weeks. *C. neoformans* was identified in 14/14 by fungal culture, 13/14 by India Ink, 13/14 by the ME Panel, 14/14 by the latex agglutination test, and 12/14 by lateral flow assay. Bacterial agents were identified in 13 patients by the ME Panel, of whom 12 were adjudicated to have confirmed bacterial meningitis, primarily *S. pneumoniae* (*n* = 9) and *L. monocytogenes* (*n* = 3); one ME-positive result was in a patient ultimately adjudicated to have tuberculous meningitis. Only three were identified by Gram stain. The ME panel identified a viral pathogen in four patients.

All cryptococcal meningitis assays performed similarly, with no significant difference in the proportion of positive outcomes (*P* = 0.89). Tuberculosis culture tests were compared (Ogawa vs LJ), and the proportions of positive outcomes were not significantly different, despite differences in sample sizes (*P* = 0.50). Comparing the same diagnostic tests performed as standard of care and for study purposes, only the Gram stain test showed a higher proportion of positive cases in the DSW (*P* = 0.02), with the remaining assays showing no statistical difference.

Regarding the DSW, for HIV-positive patients, a positive test for cryptococcal infection was the strongest predictor; if negative, the model evaluated TB status. The model achieved perfect classification in the training data but showed some overfitting in the test set, with a misclassification rate of 16.7%. For HIV-negative patients, the bacterial marker was the strongest predictor; if negative, TB status was the next key factor, with a small misclassification rate of 10%. Unlike in HIV-positive patients, cryptococcal infection did not play a significant role in classification. We did not identify bacterial meningitis among HIV-positive patients in this cohort.

In contrast, 54.2% of bacterial infections occurred in HIV-negative individuals, reinforcing the need for HIV testing as the initial step to differentiate diagnostic pathways. We did not observe bacterial meningitis among HIV-positive patients in our cohort, which is consistent with the predominance of opportunistic CNS pathogens in advanced HIV disease: among HIV-positive patients in our cohort with CD4 counts available, CD4 <200 cells/µL was documented in 84.6% (Table 3), a profile in which *Cryptococcus neoformans*, *Mycobacterium tuberculosis*, and viral opportunists predominate over pyogenic bacterial pathogens. This finding should therefore be regarded as biologically congruent with the underlying immunological state of the cohort, while acknowledging that the small absolute number limits statistical inference and may not generalize to HIV cohorts with preserved CD4 counts.

The DSW significantly outperformed the standard-of-care identification of causative agents; it detected pathogens in 43/44 (97.7%) confirmed cases compared with 26/44 (59%) identified through assays performed as standard of care at the hospital (*P* < 0.001) (Table 2).

In a per-pathogen analysis of the 44 confirmed CNS infections (Table 2), the two laboratories were 100% concordant for cryptococcal meningitis (14/14 detected by both). The DSW provided incremental detection in three categories: tuberculous meningitis, with 6/14 confirmed cases (43%) identified only by the DSW, primarily driven by systematic Xpert MTB/RIF Ultra testing; bacterial meningitis, with 8/12 confirmed cases (67%) identified only by the DSW, driven by FilmArray detection of fastidious organisms; and viral meningitis/encephalitis, with 4/4 confirmed cases (100%) identified only by the DSW as no viral assay was available in the hospital SoC menu. The DSW did not increase yield uniformly across etiologies but addresses pathogen-specific diagnostic gaps in the local SoC menu. The ME panel detected bacterial targets in 13 patients, of whom 12 were adjudicated to have bacterial meningitis; one ME-positive result was observed in a patient ultimately classified as having tuberculous meningitis.

### Clinical outcomes

Among the 93 patients with documented hospital outcome (10 remained admitted at study closure or had unknown disposition), in-hospital mortality occurred in 15 patients (16.1%), and 78 (83.9%) were discharged. Mortality varied by etiology: TBM, 5/14 (35.7%); bacterial meningitis, 3/12 (25.0%); cryptococcal meningitis, 4/14 (28.6%); viral, 0/4; non-infectious, 1/20 (5.0%); and probable infection, 2/29 (6.9%). Median length of stay was longer in confirmed infectious etiologies than in non-infectious cases. Although follow-up beyond discharge was incomplete, we did not formally evaluate antimicrobial utilization, time to appropriate therapy, or post-discharge outcomes; the DSW shortened the time to a definitive etiologic result by avoiding 4–10-week waits for solid-medium mycobacterial culture in the 13 cases in which Xpert Ultra returned a same-day result and by providing a result in <2 h in the 12 cases positive on the FilmArray ME Panel.

### Cost simulation

Among the 44 confirmed cases, 30/44 (68%) were resolved at Tier 1, 6/44 (14%) required Tier 2 (Xpert MTB/RIF Ultra), and 8/44 (18%) required Tier 3 (FilmArray). Applying the incremental tariffs in Table S1, the stopping-rule DSW cost was S/. 46,495.80 vs S/. 120,205.80 under all-tests-to-all strategy—a 61.3% reduction (saving S/. 73,710.00). Extrapolating to the full 103-patient analytical cohort (in which 39 probable and 20 non-infectious cases traversed the entire algorithm without a Tier-1 or Tier-2 stopping positive), simulated cost was S/. 207,680.85 with stopping rule vs S/. 281,390.85 without—a 26.2% reduction (identical absolute saving of S/. 73,710.00, distributed over the larger denominator). The savings correspond to the 36 confirmed patients (Tiers 1 + 2) who avoided downstream FilmArray testing. Sensitivity analysis (±20% variation in workflow-level distribution) preserved savings within 54.7%–69.3% (confirmed cases) and 23.4%–29.6% (full cohort), supporting robustness. Per-patient cost figures are summarized in Table S1.

## DISCUSSION

While clinical findings are essential for raising initial suspicion of meningitis and can be accompanied by specific findings that point toward a particular etiology, early identification of the causal agent is critical for selecting appropriate antimicrobial therapy, which may improve treatment outcomes, reduce mortality, and reduce long-term neurological complications (13). A DSW integrating rapid tests can significantly enhance prompt and accurate diagnoses, optimizing patient care and outcomes.

Our findings support implementing a diagnostic stewardship workflow that prioritizes targeted testing based on clinical and epidemiologic risk factors. This structured approach enhances diagnostic efficiency, has the potential to reduce unnecessary testing, and may support optimization of antimicrobial use, especially in resource-limited settings. However, a direct measurement of antimicrobial-prescribing change attributable to the DSW would require an interventional study design. The findings of our study suggest that including rapid HIV testing, in addition to standard-of-care analyses, should be a standard component of the initial round of tests. The inclusion of HIV testing will enhance the likelihood of early diagnosis where HIV and tuberculosis are highly prevalent, and patients are predisposed to serious diseases like CM and TBM.

In HIV-positive patients, assays to detect cryptococcal infection should be prioritized. Antigenic tests, such as the rapid lateral flow assay, detect cryptococcal antigens and can be used at the point of care with similar sensitivity and specificity to other diagnostic assays (ME panel, India Ink, and culture) (14, 15). Our findings support including this test for all patients with HIV infection or HIV rapid test positive presenting with subacute or chronic meningitis. Unfortunately, this assay does not provide antigen quantification and is not widely available globally (16, 17).

If the LFA test result is negative or other methods rule out CM, tuberculosis is the next most important and common pathogen to consider. For TBM, GeneXpert is the molecular diagnostic method recommended by WHO for screening patients with HIV infection (18). In a systematic review, the pooled sensitivity of Xpert Ultra was 64%, and specificity was 100%; the study did not compare efficacy between Xpert Ultra and culture (19). In this study, Xpert Ultra performed better than cultures on solid media (Ogawa and LJ). Xpert Ultra has been reported to have higher sensitivity for detecting smear-negative and HIV-associated TB than the Xpert MTB/RIF assay. It detects more RIF mutations, making Ultra the preferred method for rapid TBM diagnosis and detection of multidrug resistance (20).

Although CSF culture remains the gold standard for definitive diagnosis of many bacterial, fungal, or mycobacterial CNS infections, the prolonged time required to obtain culture results often significantly reduces its clinical utility for decision-making, especially for mycobacterial infections. Such delays usually inhibit prompt initiation of therapy, thus increasing the risk of worse outcomes or death (6). Furthermore, additional steps to improve diagnostic yield, such as centrifugation and concentration of CSF samples, increase the diagnostic yield of traditional and molecular CSF tests like the Gram stain or Xpert/Ultra MTB/RIF, which are particularly pertinent in settings with high endemicity of TBM (21, 22).

In patients without HIV infection, in whom a diagnosis is not obtained through direct examination for pathogens, the BioFire FilmArray ME panel should be considered as the next step. In low- to middle-income settings where the cost of this panel may be prohibitive, a DSW methodology could improve the performance of traditional assays. Novel molecular tests have reduced the cost of inappropriate empirical antimicrobial treatment by accelerating time to a definitive diagnosis (23, 24). A systematic review concluded that the ME panel was highly sensitive in identifying bacterial and viral causes of CNS infections in immunocompetent patients (25). A previous study in Peru of more than 500 patients with symptoms of meningitis/encephalitis using the ME panel detected a pathogen in CSF in 20% of patients. The most common etiologies included enterovirus, *Cryptococcus neoformans*, *Streptococcus pneumoniae*, *Listeria monocytogenes*, and human herpesvirus 6 (3).

A diagnostic stewardship approach using the ME panel has been associated with improved appropriate antimicrobial use among children with suspected CNS infections and reduced complications from unnecessary treatment. Multiple studies have demonstrated the cost-effectiveness of using the ME panel in BM (26, 27). Advantages include a simple setup, minimal processing steps, a low required CSF sample volume, and the detection of multiple potential pathogens in a single assay (24, 28). In addition, molecular assays, such as conventional PCR, GeneXpert, and BioFire FilmArray, are becoming increasingly available and have similar speed and diagnostic parameters as antigenic tests but often require additional laboratory equipment and resources and are more costly than traditional microbiological tests (29–31).

This study was conducted in a single hospital setting, which may introduce selection bias, and the prevalence of other etiologies may have been underestimated. The single-center nature of the study, conducted in a tertiary public hospital in Lima with high concurrent burdens of HIV and tuberculosis, limits external validity. The DSW logic is portable, but the specific test ordering priorities and pre-test probabilities should be recalibrated to local epidemiology when applied in lower-prevalence settings or in cohorts with predominantly community-acquired bacterial meningitis. Given the modest sample size (*n* = 103), the classification tree should be interpreted as a descriptive, hypothesis-generating tool to inform DSW structure rather than as a predictive model for clinical deployment; external validation in larger, multi-site cohorts is required before any predictive use.

Additionally, we did not perform a cost-effectiveness analysis, which has been a limitation of our study. Future studies incorporating multicenter data and economic evaluations are needed to refine and optimize diagnostic stewardship strategies. We acknowledge that BioFire FilmArray and Xpert MTB/RIF Ultra remain capital-intensive and may not be available across all LMIC settings. The DSW logic, however, is portable to contexts where only Tier 1 and a single targeted molecular assay exist: in our cohort, 68% of confirmed cases were detectable at Tier 1 alone, indicating that even without Tier 3 capacity, the DSW retains substantial diagnostic value while continuing to limit unnecessary testing. Prospective studies incorporating patient-centered endpoints will be needed to establish its full clinical impact.

### Conclusion

Our study demonstrates that implementing a diagnostic stewardship workflow substantially improves the detection of meningitis etiologies in a resource-limited setting. By integrating early HIV testing, rapid cryptococcal antigen detection, and targeted use of molecular assays such as Xpert Ultra and the BioFire ME Panel, the DSW achieved high pathogen identification rates and outperformed the standard diagnostic approach. The algorithm also reduces unnecessary testing and streamlines the diagnostic pathway according to patient HIV status, which is a critical determinant of etiology. These findings support incorporating structured DSW algorithms into meningitis evaluation protocols in low-resource settings to accelerate diagnosis and improve clinical decision-making; antimicrobial optimization is a plausible downstream benefit that was not directly measured. Future multicenter studies with cost-effectiveness evaluations are needed to validate and refine this model.

## Data Availability

The data sets are not publicly available because they form part of an ongoing program of analyses by the study team and contain sensitive clinical information from a vulnerable patient population collected at a single tertiary hospital, which could pose a risk of indirect re-identification. De-identified data supporting the findings of this study are available from the corresponding author upon reasonable request and following approval by the institutional ethics committee of the Hospital Nacional Dos de Mayo, subject to a data-use agreement.

## SUPPLEMENTAL MATERIAL

Table S1 spectrum.04173-25-s0001.docx Cost analysis.

ASM does not own the copyrights to Supplemental Material that may be linked to, or accessed through, an article. The authors have granted ASM a non-exclusive, world-wide license to publish the Supplemental Material files. Please contact the corresponding author directly for reuse.
