# Supplementary material for: Enhancing meningitis diagnosis: a diagnostic stewardship workflow in a resource-limited setting
Source: Microbiol Spectr. 2026 Aug 4;14(9):e04173-25. doi: 10.1128/spectrum.04173-25 (PMC13532260; doi:10.1128/spectrum.04173-25)
Supplement: Supplementary file 1 [file spectrum.04173-25-s0001.docx]

# **Supplementary Table S1**

## **Cost Matrix of the Diagnostic Stewardship Workflow per Cerebrospinal Fluid Sample**

### **Section A — Unit cost per individual diagnostic test**

| **#** | **Test** | **Unit cost (S/.)** | **Workflow Tier** | **Lab** |
| --- | --- | --- | --- | --- |
| 1 | CSF cytochemistry | 69.55 | 1 | Both |
| 2 | Latex agglutination / Lateral Flow | 76.70 | 1 | Both |
| 3 | Coagglutination, Hib/Spn/Strep B/Nm tests | 107.90 | 1 | ROE |
| 4 | India ink | 15.60 | 1 | Both |
| 5 | Adenosine deaminase | 44.85 | 1 | Both |
| 6 | Fungal culture | 54.60 | 1 | Both |
| 7 | Bacterial culture | 54.60 | 1 | Both |
| 8 | Blood-culture bottle | 58.50 | 1 | Both |
| 9 | Mycobacterial culture (Lowenstein-Jensen) | 59.15 | 1 | Both |
| 10 | Lactic dehydrogenase | 29.25 | 1 | Both |
| 11 | VDRL | 29.25 | 1 | Both |
|  | **Subtotal Tier 1 (Standard CSF panel)** | **599.95** | **1** | — |
| 12 | Xpert MTB/RIF Ultra | 507.00 | 2 | ROE |
| 13 | FilmArray ME Panel (BioFire) | 1,625.00 | 3 | ROE |
|  | **Maximum cumulative cost per patient (Tier 1+2+3)** | **2,731.95** | — | — |

### **Section B — Cumulative cost per workflow tier**

| **Workflow level** | **Tests included** | **Cumulative cost (S/.) per patient** |
| --- | --- | --- |
| **Tier 1** | Standard CSF panel (11 tests) | 599.95 |
| **Tier 2** | Tier 1 + Xpert MTB/RIF Ultra | 1,106.95 |
| **Tier 3** | Tier 2 + FilmArray ME Panel | 2,731.95 |

### **Section C — Cost simulation among patients with confirmed infectious meningitis**

#### **C.1. Sequential workflow with stopping rule (Diagnostic Stewardship Workflow)**

| **Workflow tier reached** | **Patients (Q)** | **Cost per patient (S/. P)** | **Subtotal (S/. P×Q)** |
| --- | --- | --- | --- |
| Tier 1 | 30 | 599.95 | 17,998.50 |
| Tier 2 | 6 | 1,106.95 | 6,641.70 |
| Tier 3 | 8 | 2,731.95 | 21,855.60 |
| Total — DSW (stepwise) | 44 | — | 46,495.80 |

#### **C.2. All-tests-to-all approach (Comprehensive testing without stopping rule)**

| **Approach** | **Patients** | **Cost per patient (S/.)** | **Total (S/.)** |
| --- | --- | --- | --- |
| All tier 1+2+3 tests on every patient | 44 | 2,731.95 | 120,205.80 |

#### **C.3. Comparative summary**

| **Strategy** | **Total cost (S/.)** | **Relative to the DSW baseline** | **Cost saved (S/.)** | **% reduction** |
| --- | --- | --- | --- | --- |
| DSW (stepwise) | 46,495.80 | 100% | — | — |
| All-tests-to-all | 120,205.80 | 258% | -73,710.00 | — |
| DSW vs All-tests-to-all | — | — | 73,710 saved | 61.3% |

### **Section D — Extrapolation to the full cohort (n = 44 confirmed infections in 103 patients)**

| **Tier** | **n with stopping** | **n without stopping** | **Unit cost** | **Cost DSW** | **Cost all-to-all** |
| --- | --- | --- | --- | --- | --- |
| **Tier 1 (All)** | **103** | **103** | **S/. 599.95** | **S/. 61,794.85** | **S/. 61,794.85** |
| **Tier 2 (after Tier-1-neg)** | **73 (103−30)** | **103** | **S/. 507.00** | **S/. 37,011.00** | **S/. 52,221.00** |
| **Tier 3 (after Tier-2-neg)** | **67 (73−6)** | **103** | **S/. 1,625.00** | **S/. 108,875.00** | **S/. 167,375.00** |
| **TOTAL** | **—** | **—** | **—** | **S/. 207,680.85** | **S/. 281,390.85** |

### Saving: S/. 73,710.00 = **26.2%**

**Sensitivity-analysis range**

| **Scenario (distribution at Tiers 1/2/3)** | **44 confirmed: DSW cost** | **Saving** | **103 cohort: DSW cost** | **Saving** |
| --- | --- | --- | --- | --- |
| Baseline (30/6/8) | S/. 46,495.80 | **61.3%** | S/. 207,680.85 | **26.2%** |
| +20% Tier 1 (36/4/4) | S/. 36,953.80 | 69.3% | S/. 198,138.85 | 29.6% |
| −20% Tier 1 (24/9/11) | S/. 54,412.80 | 54.7% | S/. 215,597.85 | 23.4% |
| **Sensitivity range** | — | **54.7 – 69.3%** | — | **23.4 – 29.6%** |
